# Supplementary material for: Male Sexual Preference for Female Swimming Activity in the Guppy (Poecilia reticulata)
Source: Biology (Basel). 2021 Feb 12;10(2):147. doi: 10.3390/biology10020147 (PMC7918064; doi:10.3390/biology10020147)

Picture for Animation 1

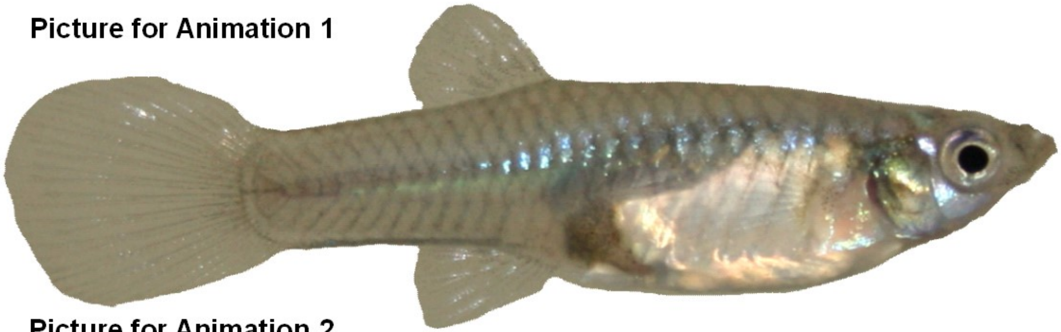

Picture for Animation 2

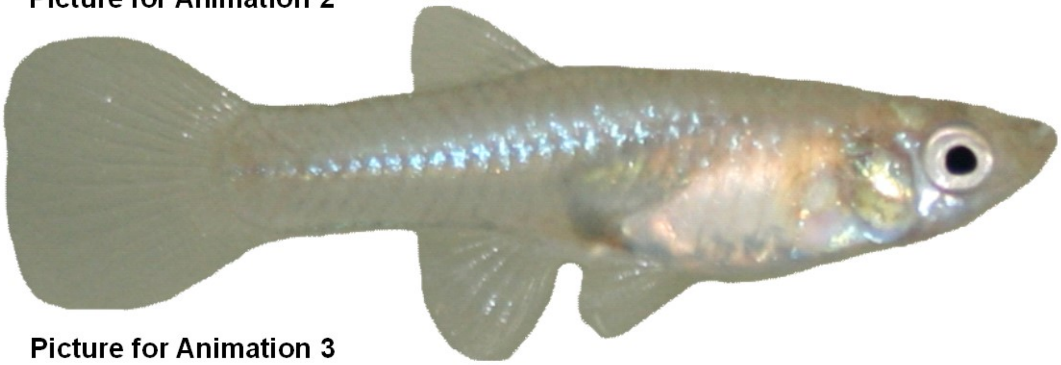

Picture for Animation 3

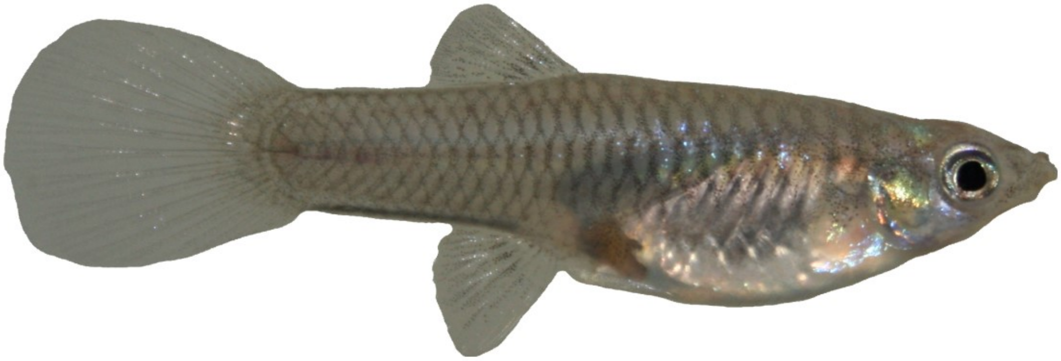

Picture for Animation 4

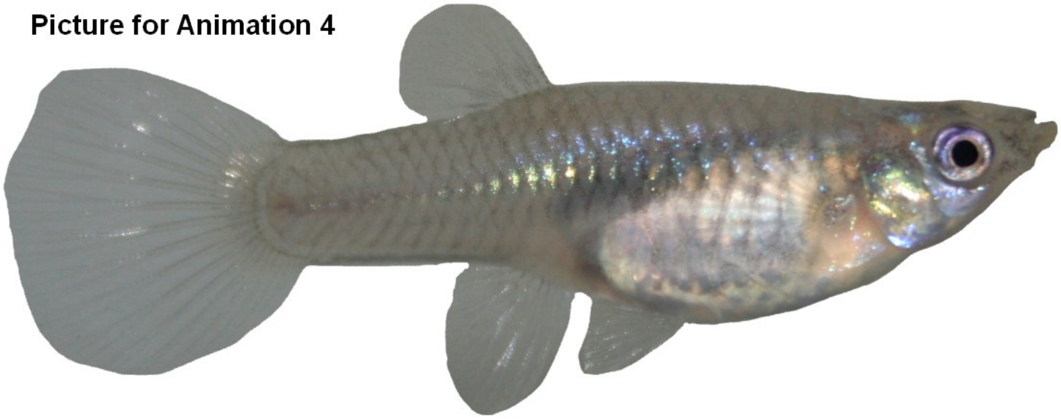

Picture for Animation 5

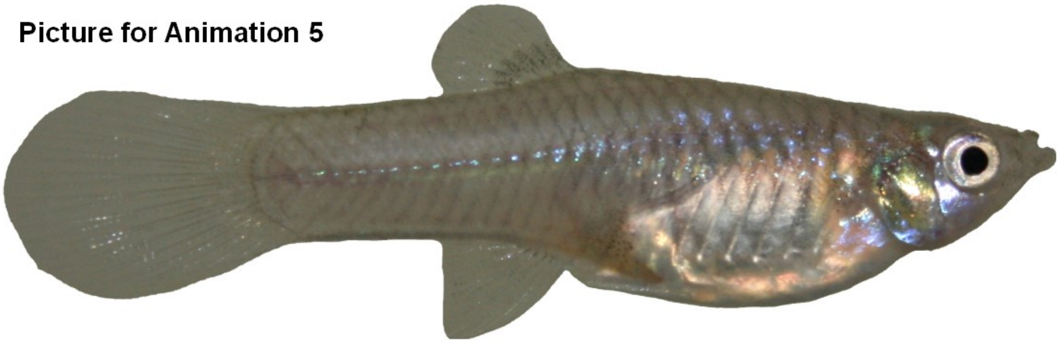

Supplement: Supplementary file 1 [file biology-10-00147-s001.zip › Figure_S1.pdf]
